# Deformable titanium for acetabular revision surgery: a proof of concept

**DOI:** 10.1186/s41205-023-00177-9

**Published:** 2023-06-09

**Authors:** J. Magré, K. Willemsen, H. M. A. Kolken, A. A. Zadpoor, H. C. Vogely, B. C. H. van der Wal, H. Weinans

**Affiliations:** 1 Department of Orthopedics, University Medical Center Utrecht, Heidelberglaan 100, 3584 CX Utrecht, The Netherlands; 2 3D Lab, Division of Surgical Specialties, University Medical Center Utrecht, 3584 CX Utrecht, The Netherlands; 3 Department of Biomechanical Engineering, Delft University of Technology, 2628 CD Delft, The Netherlands

**Keywords:** 3D printing, Implant fixation, Implant revision, Biomechanics, Reconstruction, Deformable titanium, Acetabular cup

## Abstract

Custom-made triflange acetabular implants are increasingly used in complex revision surgery where supporting bone stock is diminished. In most cases these triflange cups induce stress-shielding. A new concept for the triflange is introduced that uses deformable porous titanium to redirect forces from the acetabular rim to the bone stock behind the implant and thereby reduces further stress-shielding. This concept is tested for deformability and primary stability.

Three different designs of highly porous titanium cylinders were tested under compression to determine their mechanical properties. The most promising design was used to design five acetabular implants either by incorporating a deformable layer at the back of the implant or by adding a separate generic deformable mesh behind the implant. All implants were inserted into sawbones with acetabular defects followed by a cyclic compression test of 1800N for 1000 cycles.

The design with a cell size of 4 mm and 0.2 mm strut thickness performed the best and was applied for the design of the acetabular implants. An immediate primary fixation was realized in all three implants with an incorporated deformable layer. One of the two implants with a separate deformable mesh needed fixation with screws. Cyclic tests revealed an average additional implant subsidence of 0.25 mm that occurred in the first 1000 cycles with minimal further subsidence thereafter.

It is possible to realize primary implant fixation and stability in simulated large acetabular revision surgery using a deformable titanium layer behind the cup. Additional research is needed for further implementation of such implants in the clinic.

**Supplementary Information:**

The online version contains supplementary material available at 10.1186/s41205-023-00177-9.

## Introduction

A growing number of primary total hip arthroplasties are carried out annually due to the rising life expectancy. This, in turn, results in more revision surgeries, particularly in patients that had surgery at a relatively young age. In the United States alone revision arthroplasties are expected to grow by 137% by 2030 [1]. The main reason for revision surgeries is aseptic loosening of the implant (55%) [2]. For long-term fixation of uncemented acetabular cups bony ingrowth is required, which relies on the initial stability after implantation. There are a number of reasons that lead to insufficient fixation: i) micromotion at the bone-implant interface that results in fibrous tissue formation instead of bone ingrowth; ii) polyethylene wear particles that initiate an inflammatory response and subsequent bone loss; iii) infection related inflammation and bone loss; and iv) relative mechanically unloading (stress-shielding) of the bone stock under the acetabular implant [3, 4].

Numerous surgical techniques and implants are available to increase the success of revision total hip arthroplasties, including structural allografts, (non-)cemented hemispherical cups, oblong cups, jumbo cups, anti-protrusio cages, and Trabecular Metal augments and shells [5–9]. However, not every revision method and/or procedure is suitable for every large acetabular defect, resulting in high re-revision rates due to the (recurrent) loosening or implant migration of the acetabular cup [10].

Due to the high failure rates associated with revision cases, complex acetabular defects (*e.g.*, Paprosky 3A/3B) are nowadays often treated with a patients-specific implant that precisely follows the actual shape of the acetabular cavity [11–14]. They often mechanically rely on three flanges that are screwed onto the cortices of the ilium, ischium, and pubis to obtain initial stability directly after implantation (triflange cups). However, the design is challenging as a perfect fit can never be reached due to complex shaped bone defects and segmentation errors due to imaging artefacts. A slight oversize of the stiff cup will lead to a bad fit with local overload and chances of instability with subsequent wiggling and micromotion. Therefore, most triflange revision cups are slightly undersized and in the deep central zone of the acetabulum there is no bone-implant contact. As a result, all loads are transferred via the flanges, leading to stress-shielding of the trabecular bone located underneath the implanted cup [15–17]. This lack of mechanical stimulus could eventually lead to even more bone resorption and might further destabilize the fixation of the acetabular cup and/or the stability of the entire pelvis [18, 19].

The abovementioned shortcomings of the current designs motivated us to develop alternative designs for the acetabular component that helps achieve a more natural stress distribution, with limiting stress-shielding while providing sufficient primary stability. Modern additive manufacturing technologies, including selective laser melting (SLM), can be used to manufacture patient-specific implants from ductile metals, such as commercially pure titanium (Grade 1) [20]. Pure titanium shows a mechanical behaviour that is somewhat similar to tantalum and outperforms its alloyed counterparts (*e.g.*, Ti-6Al-4 V) in terms of the normalized high cycle fatigue strength. These properties make it a suitable material for cyclically loaded implants such as acetabular components [21]. With the current 3D printing techniques, structures as small as 200 μm can be manufactured at a reasonable accuracy, which present opportunities for highly porous structures that undergo substantial plastic deformation under compression. Incorporating such a deformable zone in acetabular implants renders the under-sizing of the implant unnecessary, as the implant will ‘deform’ to match the patient’s anatomy. Moreover, these porous (*i.e.*, trabecular bone-like) structures generally expand laterally in response to axial compression, filling up complicated bone defects during insertion of the implant [22], thereby creating substantial implant-bone interface surface and primary stability.

In this study, we will test the feasibility of the abovementioned approach. The mechanical properties of highly porous pure titanium Body Centered Cubic (BCC) structures are examined in terms of stiffness, strength, plastic deformation and its capability to precisely fill an acetabular bone defect during insertion. In previous studies this type of unit cell exhibited a low yield stress and high ductility with a large lateral expansion. It is, therefore, expected to exhibit promising space-filling properties too [22]. In this study, we aim to alter the porosity such that elastic and plastic deformation of a porous deformable cup will fill the space of the acetabular defect and thereby create fixation and stability. We also evaluated the subsidence after insertion of such deformable acetabular implant under cyclic loading.

## Methods

### Study outline

**Fig. 1:**
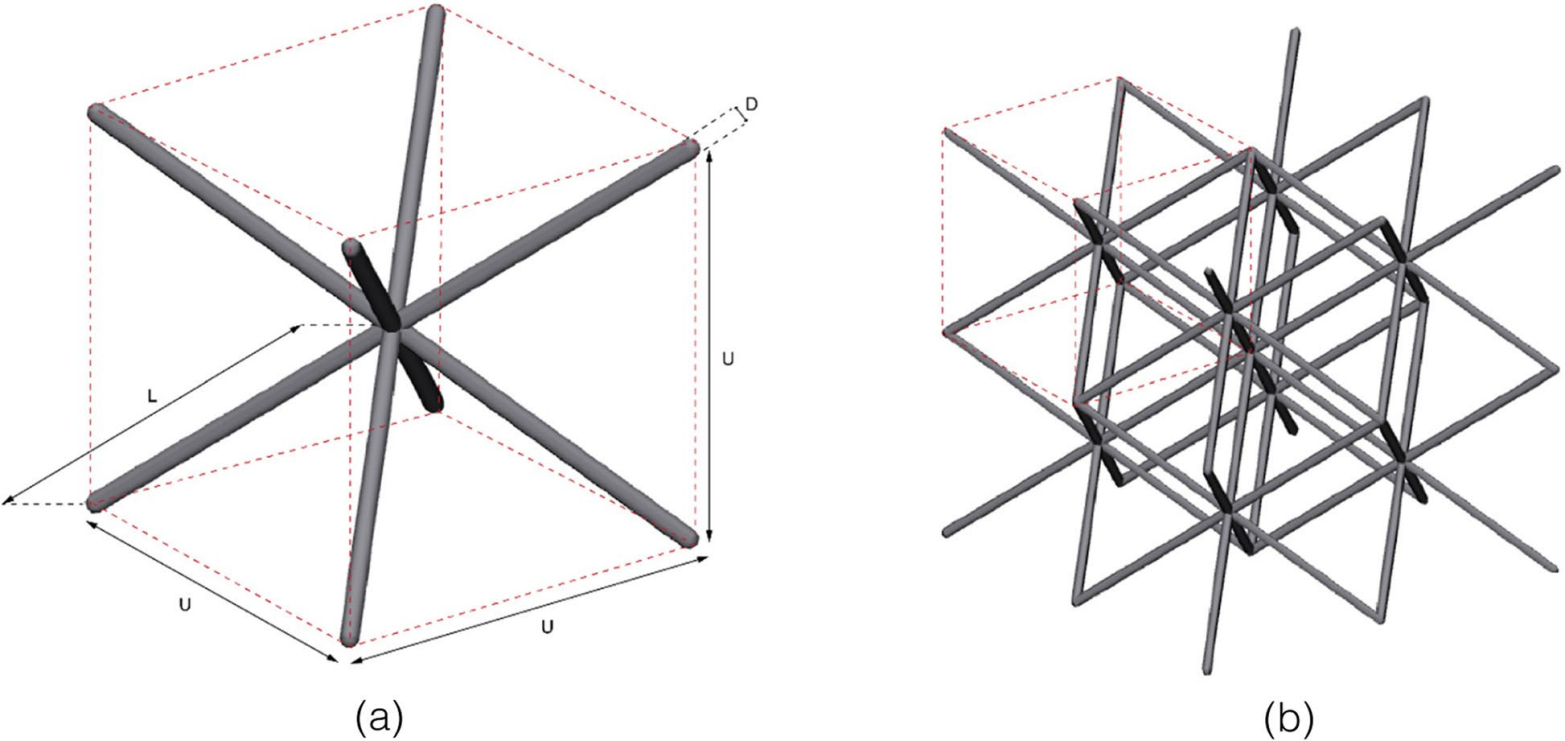
**a** The design of the body centred cubic (BCC) unit cell with the representation of the strut length (L), strut diameter (D), and unit cell size (U). **b** A 2 by 2 by 2 units porous structure consisting of 8 BCC unit cells

This study contains three parts. First, the mechanical properties of porous structures designed with different dimensions of the BCC unit cell (Fig. 1) were determined with compression tests on cylinders with varying porosities and strut thicknesses. Based on the results of the first part, one of the unit cell dimensions was chosen for the design of custom-made acetabulum implants, which were then inserted in the acetabulum of bone mimicking Sawbone pelvises (Sawbones, Limhamn, Sweden) with systematically created acetabular defects to evaluate the space-filling potential of the deformable titanium. Finally, the inserted implants were cyclically loaded to measure their post-insertion migration to indicate primary stability.

### Cylindrical samples test

**Fig. 2:**
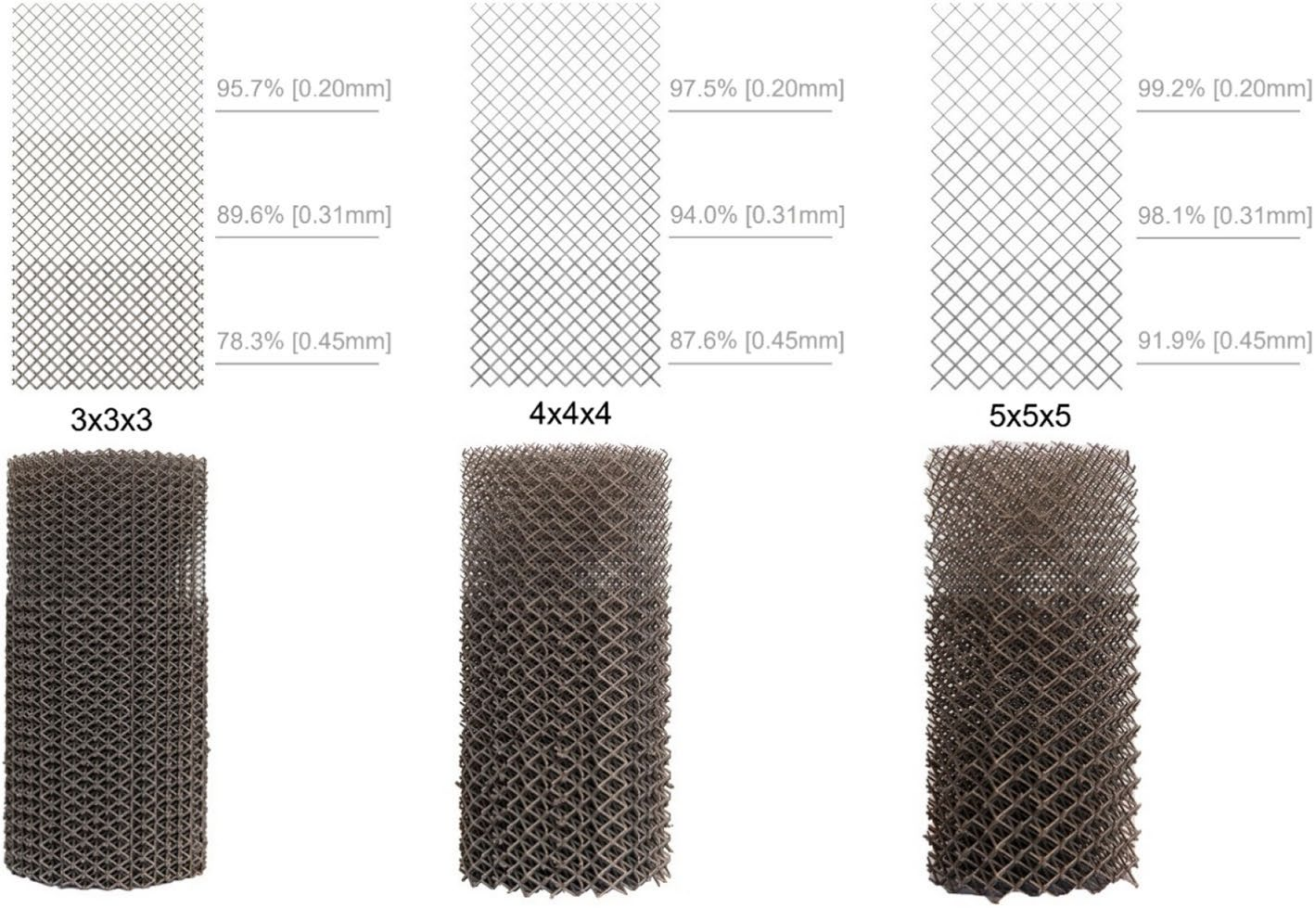
The design of the graded porous cylinders. The top images show a schematic representation of the design, the bottom images are photographs of the corresponding printed samples. Unit cell dimensions: left = 3 × 3 × 3 mm, middle = 4 × 4 × 4 mm, right = 5 × 5 × 5 mm. The porosity gradient is obtained by adjusting the strut thickness in the specimens (from top to bottom: 0.20 mm, 0.31 mm, and 0.45 mm)

Three porous cylindrical samples were designed using 3-Matic (version 13.0, Materialise NV, Leuven, Belgium) (Fig. 1). The samples were 81 mm in height and had a diameter of 40 mm. All samples featured a BCC unit cell infill, using a different unit cell size for each design [23]. The cylinders were divided into three equal sections of 27 mm (Fig. 2) with each a different strut thickness to create a graded porosity (percentage of solid volume).

The pure titanium (Grade 1) specimens were manufactured with SLM using a ProX DMP 320 (3D Systems, Leuven, Belgium) machine [24]. All specimens were tested as-manufactured without any additional post-processing. Three specimens of each design were produced (nine specimens in total).

Each specimen was subjected to a static compression test between two compression plates on a Lloyd LS 5 universal test machine (Amatek, Berwyn, United States) with a 5 kN load cell and at a rate of 2 mm/min up to a force of 4.9 kN. All the cylindrical specimens were tested in the same manner and a video recording was made of the compression test of each specimen type.

The compressive yield strengths was determined from the stress–strain curves of the cylindrical specimens using the 0.2% offset method (ISO 13314–2011) as was done by Huang et al*.* for porous structures [25]. The stress (*σ*) was calculated by dividing the applied force by the initial cross-sectional area using the (apparent) full 40 mm diameter. The strain (*ε*) was defined as the displacement divided by the original sample height per Sect. (27 mm). The elastic moduli were calculated from the stiffest part of the slope of the stress–strain curves in the linear elastic regions of the subsequent parts of the stress–strain curve.

### Deformable acetabular implant

**Fig. 3:**
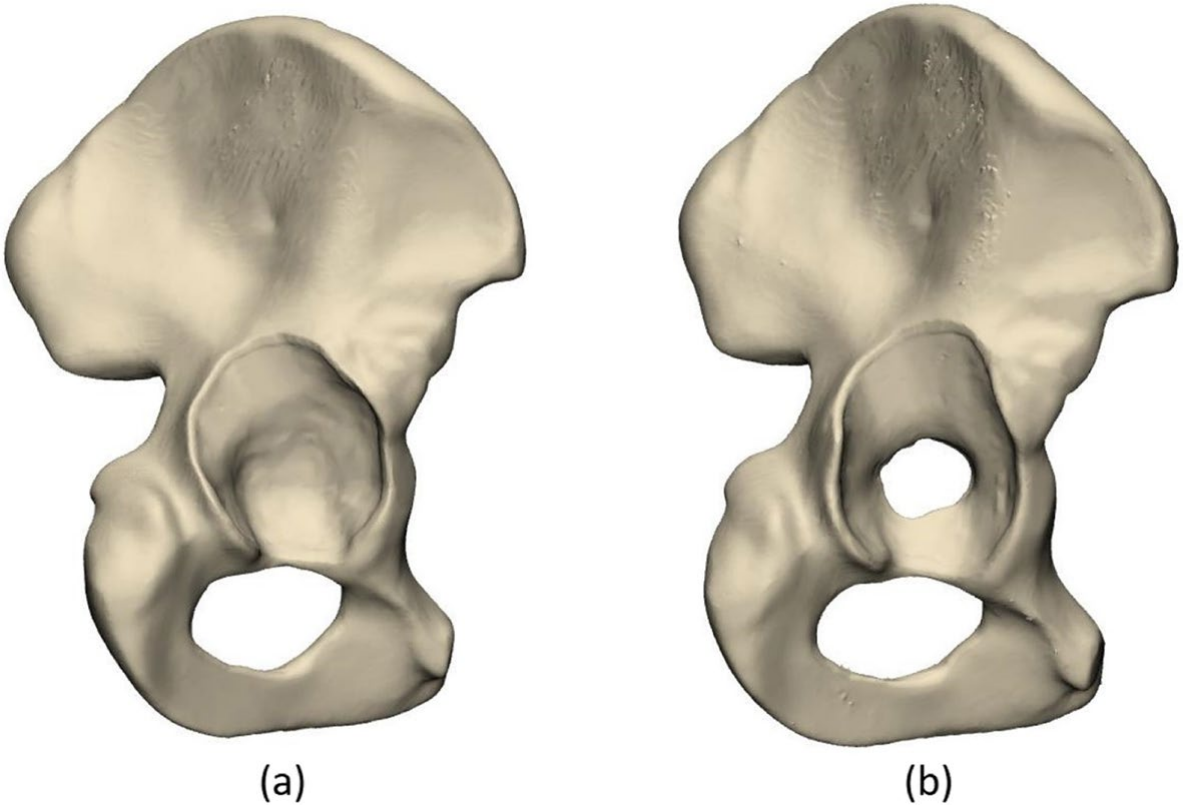
The images of the acetabular defects created in the Sawbones hemipelves. **a** acetabular defect type 1 with an intact medial wall, **b** acetabular defect type 2, with a protruded medial wall

Acetabular defects were systematically created into five right-sided biomechanical composite hemipelvis Sawbones (Hemi-Pelvis, 4th Generation, Composite, 10 PCF Solid Foam Core, Large, Sawbones, Limhamn, Sweden). The bone phantoms that were used have been specifically designed to mimic the material properties of native bone, simulating trabecular bone using a foam and a short fibre filled epoxy to simulate cortical bone [26–28]. In all five hemipelves, simplified acetabular defects were created based on the Paprosky acetabular defect classification system [12, 29]. Two types of acetabular defects were created the hemipelves (Fig. 3).

After defect creation, a clinical computed tomography (CT) scan was made of all hemipelves with a 0.8 mm slice thickness (80 kV, 280mAs, IQon Spectral, Philips, Amsterdam, Netherlands). The DICOM files were imported and segmented in Mimics (version 23.0, Materialise, Leuven, Belgium) to create three-dimensional computer models of the acetabular defects.

**Fig. 4:**
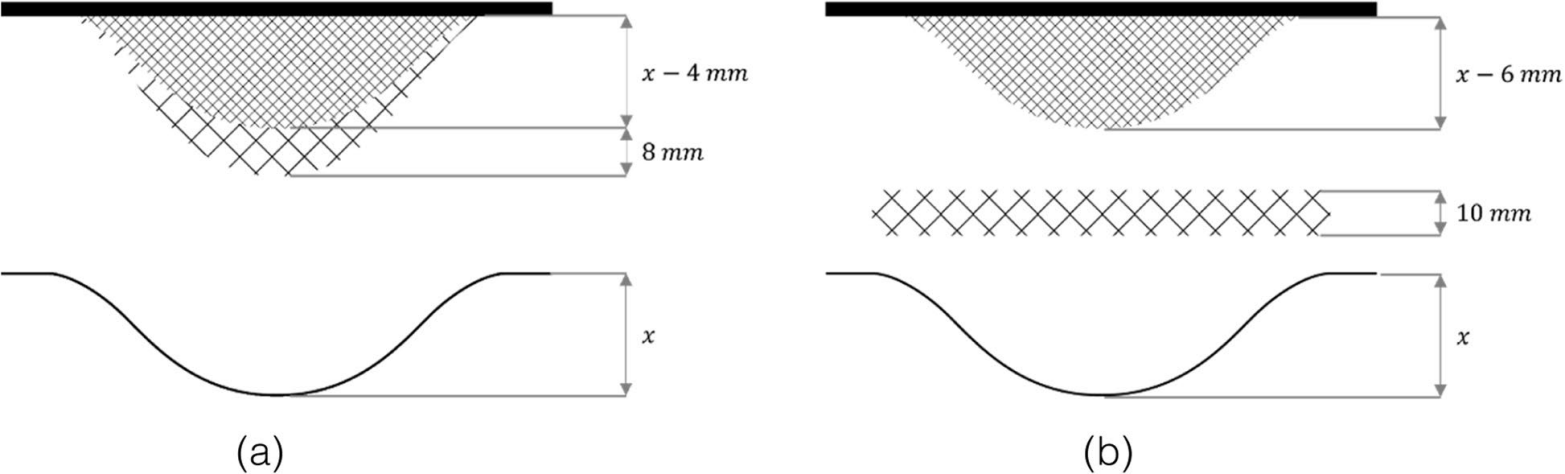
Schematic representation of the two design concepts. **a** Concept 1 features a low-porous layer which is 4 mm undersized in the direction of insertion. The highly porous deformable layer is 8 mm oversized in the direction of insertion, resulting in 4 mm oversized implants. **b** Concept 2 features a low-porous layer which is 6 mm undersized in the direction of insertion. This concept requires a separate deformable mesh with a thickness off 10 mm which also results in 4 mm oversized implants. $\mathrm{x}$ = depth of the defect in the direction of implant insertion

Five unique ‘case-specific’ acetabular cups were designed using 3-Matic (Version 15.0, Materialise, Leuven, Belgium) based on their respective 3D models of the Sawbone hemipelves. These cups can be divided into two main types: acetabular cups without flanges (flush) and acetabular triflange cups (Table 1). The triflange cups featured two design concepts: concept 1 features an incorporated oversized deformable titanium layer (Fig. 4a) and concept 2 is an undersized version that requires a separate deformable mesh being placed underneath the cup (Fig. 4b). These generic meshes were designed in SolidWorks (version 2017, Dassault Systems, France). Both concepts were designed to be oversized in direction of insertion in comparison to the existing defect (Fig. 4).

**Table 1:** Overview of the acetabular defects with their respective implant design and specifications

Acetabular cup	Defect type	Triflange/ flush	Deformable Type	Unit cell size [mm]	Strut thickness
1	Type 1	Flush	Incorporated	4 × 4x4	0.2
				4 × 4x4	0.5
2	Type 2	Triflange	Incorporated	1.5 × 1.5x1.5	0.5
			Separate mesh	4 × 4x4	0.2
3	Type 3	Flush	Incorporated	4 × 4x4	0.2
				4 × 4x4	0.5
4	Type 4	Triflange	Incorporated	2.5 × 2.5x2.5	0.5
			Separate mesh	4 × 4x4	0.2
5	Type 5	Triflange	Incorporated	4 × 4x4	0.2
				4 × 4x4	0.5

The porous layers of the cups are made based on the 4 × 4x4mm BCC porous structures. The porous structures were oriented in the direction of cup insertion, thereby facilitating lateral expansion of the BCC unit cell under compression [30].

**Fig. 5:**
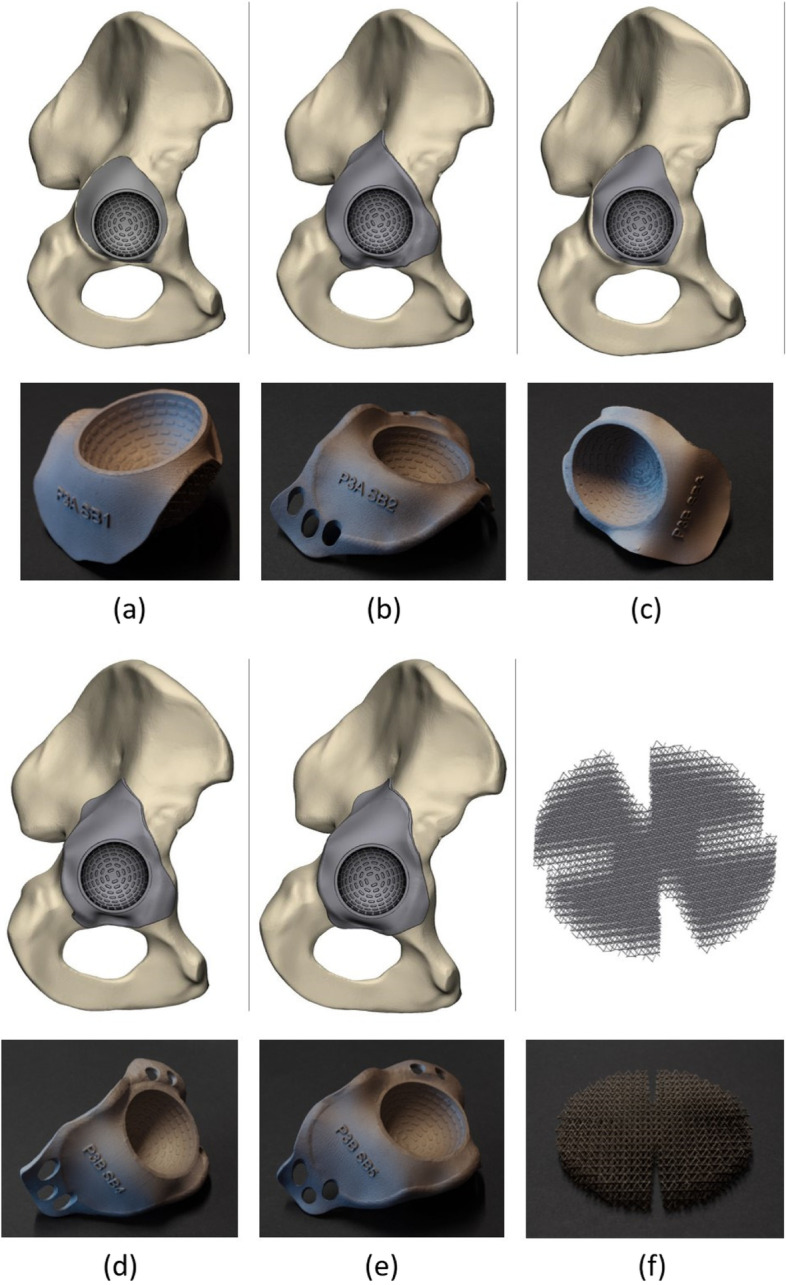
CAD models (top) and photographs (bottom) of the manufactured acetabular cups. **a** implant 1; flush design with incorporated deformable titanium layer. **b ** implant 2; triflange design which requires a separate deformable titanium mesh. **c** implant 3; flush design with incorporated deformable titanium layer **d** implant 4; triflange design which requires a separate deformable titanium mesh **e** implant 5; triflange design with incorporated deformable titanium layer **f** circular deformable titanium mesh

The incorporated porous structures of cup 1, 3, and 5 consisted of a low-porous layer with a strut thickness of 0.5 mm, the oversized deformable layer featured a strut thickness of 0.2 mm (Fig. 5a, c and e). Alternatively, cups 2 and 4 undersized incorporated low porous structure with a strut thickness of 0.5 mm (Fig. 5b and d). Two types of meshes were designed for spherical and elongated bony defects. Both highly porous mesh types were produced in three diameters: 53 mm, 63 mm, and 73 mm with a strut thickness of 0.2 mm. (Fig. 5f).

As a substitute for clinically used polyethylene liners, polyamide liners were designed to be cemented into the acetabular cups. These liners, with a thickness of 6 mm, accommodated a 28 mm femoral head for cyclic testing.

All implants were produced the same way as the cylindrical specimen. The substitute liners were produced using selective laser sintering (SLS) out of nylon (polyamide 12) (Oceanz, Ede, Netherlands).

The acetabular cups were inserted into their corresponding Sawbones acetabula by two senior orthopaedic surgeons at UMC Utrecht. The acetabular cups with attached deformable layer (1, 3, 5) were inserted directly into their position using an inserter and hammer from a standard hip insertion set (Zimmer Biomet). For the other two cups (2, 4), a separate mesh was selected by the surgeon and pressed into the acetabulum defect before the implant was inserted on top. After insertion of the acetabular implants, the liners were cemented into the cups using a bone cement (Zimmer Biomet Refobacin R®) [31].

The deformation of the deformable layer after insertion was assessed by making a new CT scan and segmenting the deformed acetabular implant from the CT using Mimics. The non-deformable solid flange of the segmented model was registered onto the original computer-aided design (CAD) model using a point-to-point registration method in 3-Matic in order to make a detailed analyses of the deformation of the deformable titanium layer.

### Cyclic loading

**Fig. 6:**
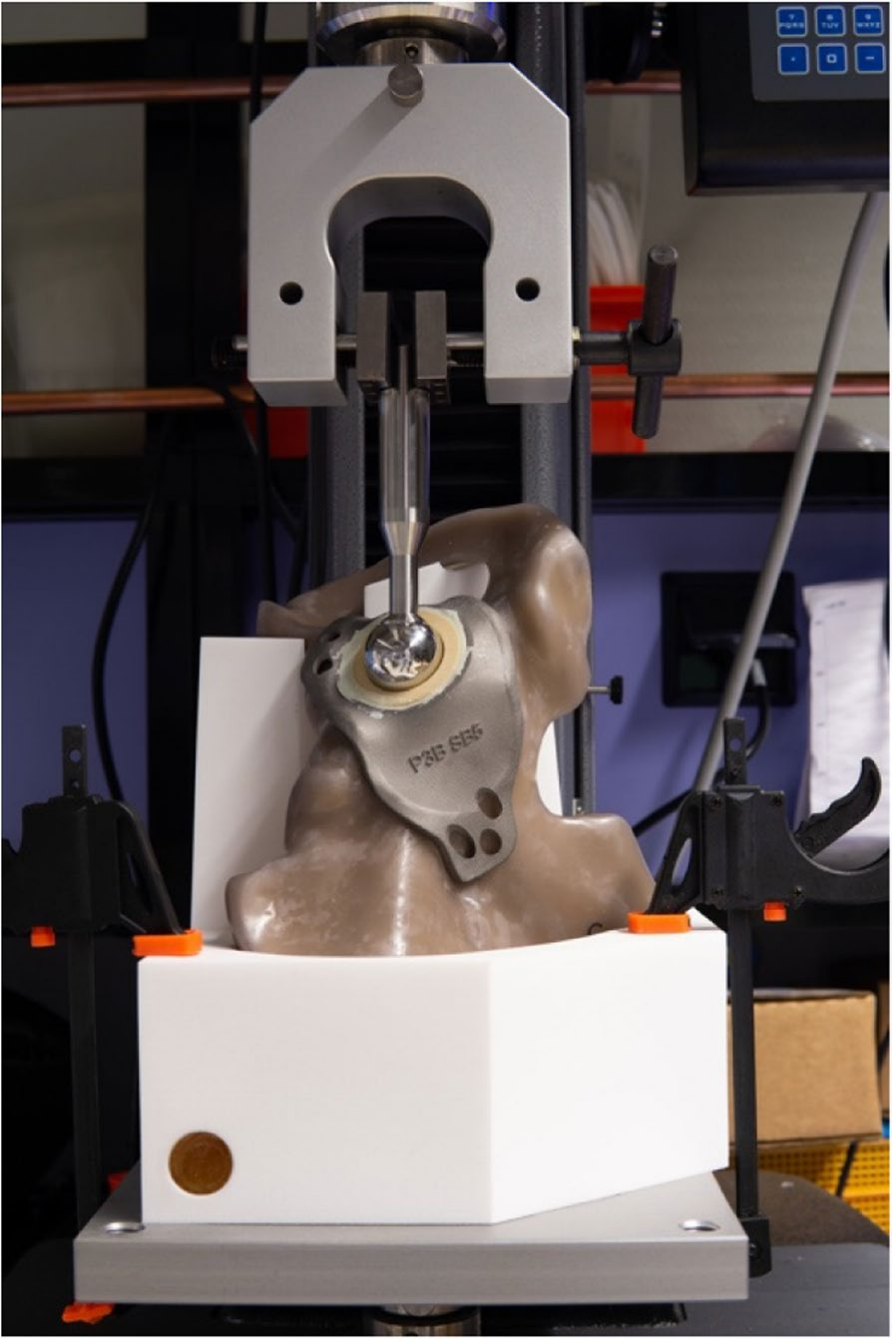
The compression testing machine setup used for the cyclic tests of the acetabular cups with the Sawbone positioned in its mould and the 28 mm femoral head positioned in the substitute liner

Cyclic tests were performed on a Lloyd LS 5 universal testing machine (Amatek, Berwyn, United States) to evaluate implant migration caused by cyclic loading. The hemipelves were positioned in an epoxy resin-filled (Polyservice, Amsterdam, Netherlands) negative mould of the pelvis that enabled a femoral head (28 mm) to deliver a cyclic force with a force vector described by Bergmann et al*.* on the cemented liner of the inserted implants (Fig. 6) [32]. All acetabular cups were tested up to 1000 cycles with a sinusoidal loading between 100 and 1800 N to simulate a gait cycle. A minimum load of 100 N was used to ensure the acetabular head did not lose contact with the liner. A maximum compressive force of 1800 N was selected based on the average peak load of gait for a body weight of 750 N [32]. The crosshead velocity of the machine was kept constant at 60 mm/min.

## Results

### Cylindrical samples test

**Fig. 7:**
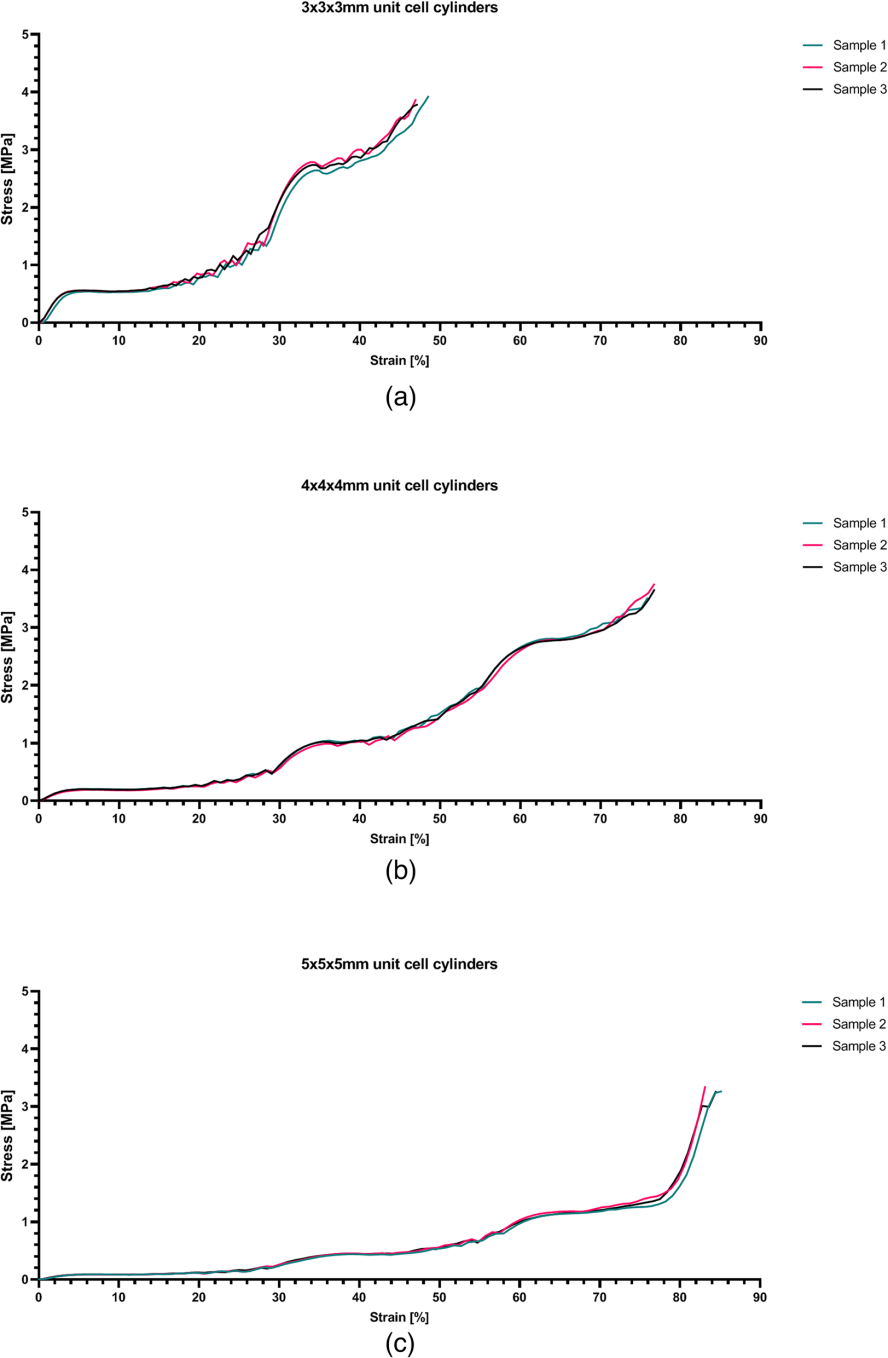
The stress–strain curves recorded during the compression tests of the cylindrical specimens. **a** The 3 × 3 × 3 mm samples, **b** 4 × 4 × 4 mm samples and **c** 5 × 5 × 5 mm all show multiple plateau regions

The smaller unit cell size specimens (*e.g.*, 3 × 3 × 3 mm) resulted in a stiffer and stronger structure than those with a larger unit cell size (*e.g.*, 5 × 5 × 5 mm). Three linear elastic sections and three plateau regions (plastic deformation) can clearly be differentiated in the case of the specimens with the 4 × 4 × 4 and 5 × 5 × 5 mm unit cell dimensions (Fig. 7). The stress–strain plot of the 3 × 3 × 3 mm specimens does not show three, but two linear elastic sections as the maximum of the 5 kN could not reach the yield point of the strongest part of this specimen with the 450 microns strut thickness (see Fig. 7a). From the stress–strain plot the apparent values of the compressive yield stress and the Youngs modulus of elasticity can be estimated for each section (Table 2).

**Table 2:** The mechanical and morphological properties of the CAD designs of the cylindrical specimens

Unit cell size [mm]	Strut thickness [mm]	Porosity %	Estimated Elastic modulus [MPa]	Compressive yield strength [MPa]	Strut ratio
3 × 3 × 3	0.20	95.7	0.224 ± 0.013	0.499 ± 0.014	12.990
3 × 3 × 3	0.31	89.6	0.380 ± 0.037	2.368 ± 0.090	8.381
3 × 3 × 3	0.45	78.3	-	-	-
4 × 4 × 4	0.20	97.5	0.066 ± 0.007	0.168 ± 0.006	17.321
4 × 4 × 4	0.31	94.0	0.145 ± 0.006	0.857 ± 0.030	11.175
4 × 4 × 4	0.45	87.6	0.190 ± 0.017	2.462 ± 0.043	7.698
5 × 5 × 5	0.20	99.2	0.026 ± 0.001	0.076 ± 0.007	21.651
5 × 5 × 5	0.31	98.1	0.039 ± 0.008	0.383 ± 0.030	13.968
5 × 5 × 5	0.45	91.9	0.087 ± 0.012	1.029 ± 0.021	9.623

**Fig. 8:**
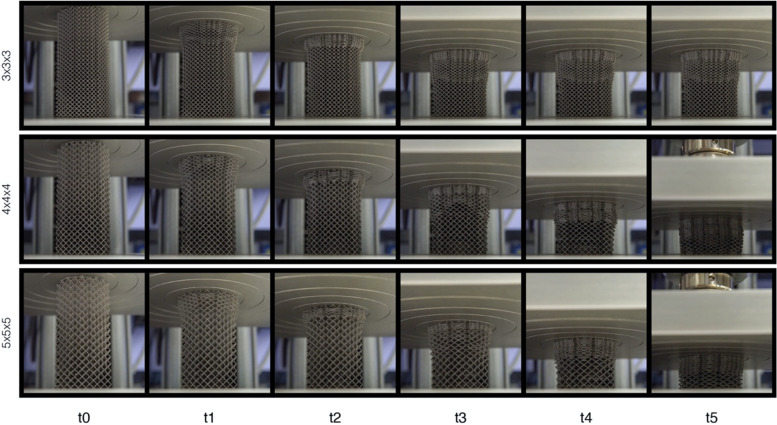
Compression of the cylindrical samples at different time intervals. Top: 3 × 3 × 3 mm, middle: 4 × 4 × 4 mm and bottom: 5 × 5 × 5 mm. At t5 the maximum compression force of 5 kN is reached for the 4 × 4 × 4 mm and 5 × 5 × 5 mm samples. Note that at t3 a load of 5 kN is reached for the 3 × 3 × 3 mm sample. Video recordings of these tests can be found in the (supplementary data) Additional files 1, 2 and 3.

All specimens deformed significantly under compression (Fig. 8). Those with largest unit cell sizes (*i.e.*, 5 × 5 × 5 mm) deformed the most. The height of the 3 × 3 × 3 mm specimens decreased 41.6% on average. For the 4 × 4 × 4 mm and 5 × 5 × 5 mm specimens, the decrease in the height was 71.1% and 79.2%, respectively. The average lateral expansion of the specimens at the fully deformed sections (end point) was 16.8% for the 3 × 3 × 3 mm specimens, 16.8% for the 4 × 4 × 4 mm specimens, and 17% for the 5 × 5 × 5 mm specimens.

**Fig. 9:**
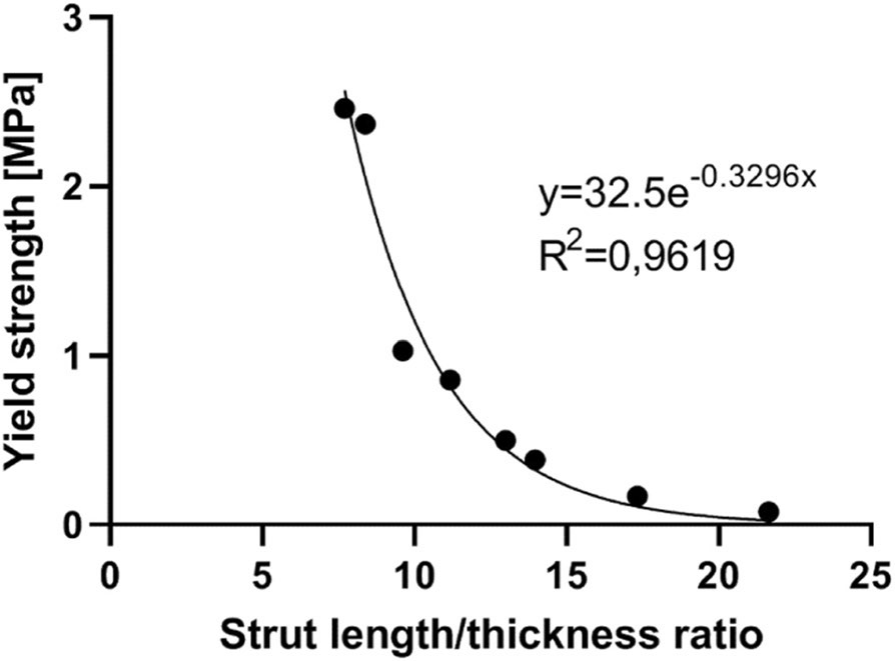
Strut length/thickness ratio plotted against the yield strength of the corresponding porous structure

The yield strength of the porous structure was an exponential function of the ratio of the strut length to the strut thickness (Fig. 9).

### Deformable acetabular implant

All five acetabular cups were implanted into their specific Sawbone defects (Table 1). Subjectively none of the surgeons felt that any of the cups required a larger impact force to insert than a regular hemispherical acetabular cup. For the undersized cups, the surgeons decided on a circular 53 mm deformable mesh (implant 2) and an elongated 53 mm mesh (implant 4). After insertion, no step-offs were observed for cups 1 and 3, which were flush with the defect border and no gaps were observed between the bone and the flanges of cups 2,4, and 5. After insertion, 4 out of 5 implants (1, 2, 3, and 5) were firmly in place and not manually removable. However, in the case of implant 4, fixation screws were needed to provide initial implant stability. Additionally, some broken titanium struts were observed as they fell through the defect in the medial wall.

**Fig. 10:**
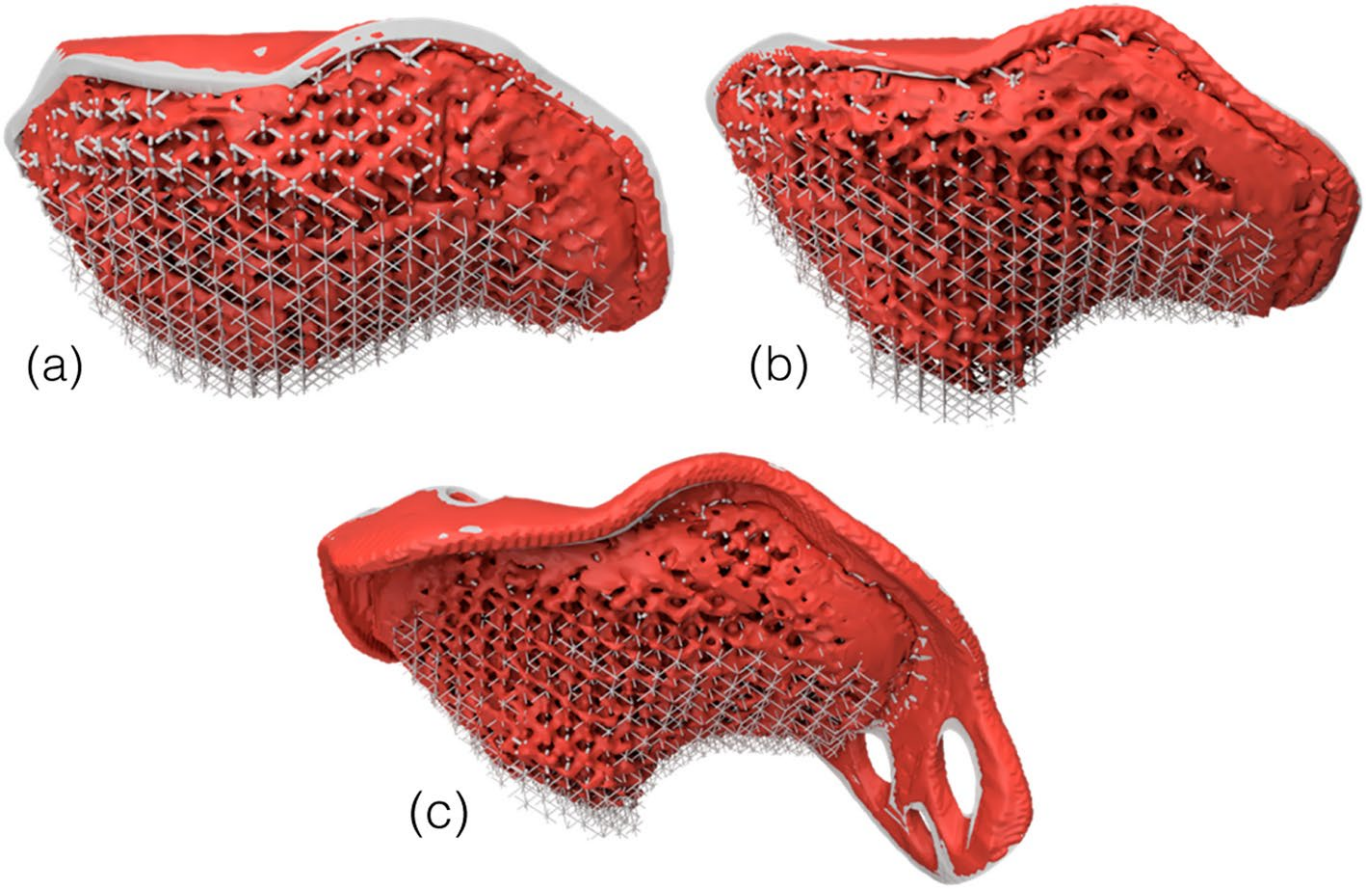
The rendered images of the CT-CAD registrations. **a** implant 1, **b** implant 3, **c** implant 5. The red part is the segmented cup after insertion, the grey colored part is the original CAD file

The post-insertion CT model of the acetabular cup was registered on the original design and showed a volumetric reduction of the deformable layer in all three acetabular cups with an attached deformable layer (Fig. 10). All cups decreased in volume after implantation within a range of 2.7 to 7.5% (see Table 3).

**Table 3:** The volumes and volume reductions of the implants before and after insertion of the acetabular cups

Acetabular cup	Volume CAD (cm^3^)	Volume CT (cm^3^)	Volume reduction (cm^3^)
1	61.98	58.68	3.3
3	65.99	61.03	4.97
5	85.60	83.26	2.35

### Cyclic loading

**Fig. 11:**
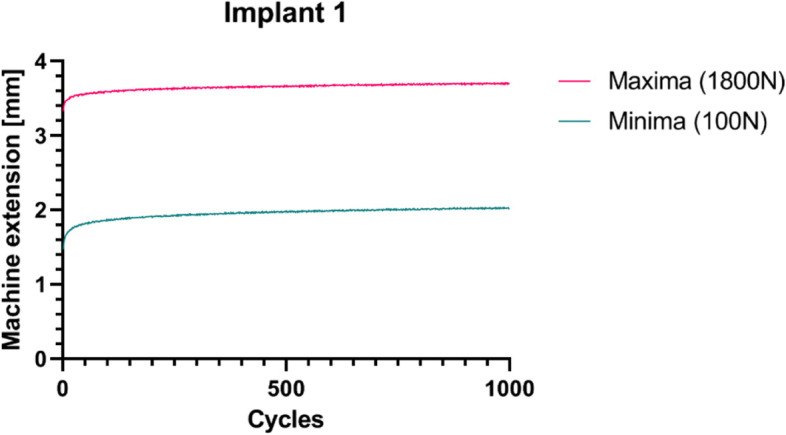
The cycles-extension curves for the cyclic test of implant 1. The extension needed to reach 1800 N during the first compression is indicated by the red line. The relaxation to 100N is shown by the blue line

Subsidence of the cups during cyclic loading after implantation was very small and reached on average up to 0.253 ± 0.034 mm, ranging from 0.1781 mm (implant 4) to 0.3793 mm (implant 1). Most of this subsidence (or migration) occurred during the first 1000 cycles of the experiment with a flattened curve approaching 1000 cycles, indicating a fully stabilized implant. A representative plot is presented in Fig. 11 while the other plots can be found in Appendix.

## Discussion

From the compression tests, we found very low yield stresses for the porous BCC structures. A compressive yield strength below 0.2 MPa was required to have a material that can be deformed during the surgical procedure. Moreover, the strut length/thickness ratio correlated with the yield strength. A ratio higher than 17 was found to be viable for deformable implants. The most optimal design with a unit cell size of 4 × 4 × 4 mm and a strut thickness of 0.2 mm (Fig. 9) was therefore incorporated into five custom-made acetabular cups. A screwless implant fixation was realized in 4 out of 5 sawbones with acetabular defects. The subsequent 1000 cycles of 1800 N compression onto the implant showed minimal additional implant migration.

The low elastic modulus and large deformation capacity of the BCC unit cell (Fig. 1) found in this study have been described in literature too [33]. The stress strain curves resulting from the compression tests with cylindrical specimens show a linear elastic region and a plateau region as expected. The three porous sections are discernible in the plots of both the 4 × 4 × 4 mm and 5 × 5 × 5 mm unit cell sizes (Fig. 7) as they plastically deform one by one. All the specimens exhibited the same type of stress–strain curve although they represent different stiffnesses and compressive strengths. However, it is apparent from the stress strain curves that the specimens with a higher porosity (thinner strut thicknesses and/or larger unit cell) showed a larger plateau region in the plastic deformation phase (Fig. 7). Elastic moduli and compressive yield strengths as low as 0.026 MPa and 0.076 MPa, respectively, were found in this experiment. It should be noted that the yield stresses can be accurately determined with our specimen, while the elastic moduli is a crude estimation based on the deformation of the entire specimen where the three porous sections are placed in series. Therefore, the elastic moduli in Table 3 represent an upper limit of the true values, as the displacement of the 1/3 region considered is less than the actual deformation used from the specimen as a whole in Fig. 7. Moreover, lateral expansion of the unit cells under uniaxial compression creates lateral friction on the compression plates, resulting in artificial stiffening of the porous structure, which compensated the above estimation. In addition, due to the graded porosity, thinner struts are connected to thicker struts at the transition, limiting lateral expansion of these thinner struts. This as well leads to slightly stiffening of the porous structure.

Usually, the material properties such as the elastic modulus and yield strength purely describe the intrinsic properties of the material and are unrelated to the morphological properties of the specimens. It is important to understand that the material properties described in these experiments describe a different concept when concerning highly porous structures. When referring to highly porous structures, such properties describe the effective macroscopic behaviour of the entire porous structure [34]. In the case of graded designs, such effective properties deviate even further from the mechanical properties of a material, as they are highly dependent on the specifics of the graded design and are by definition dependent on the specimen dimensions. Altogether the elastic moduli should be considered as estimated values as they are not precise values for the above mentioned reasons. In the current work however, we are more interested in the plastic behaviour of the material as these are key in its highly deformable and space filling properties.

The stress–strain curves show some spikes at the most deformed part of the plateau regions. These spikes could indicate the failure of struts, although no loose struts have been observed after compression. This may indicate that struts have fractured at one end only. At that point, the strut will not be bearing mechanical loads anymore and thus further weaken (and deforming) the overall structure. The specimens exhibited a layer-by-layer failure mode, starting from the highly porous top layers. This is consistent with the failure mechanism described in the literature [35, 36]. The manufacturing irregularities caused by the additive manufacturing process significantly affect the mechanical properties of the resulting porous structures [37]. The porous structures showed heterogeneous behaviour while, at the strut level, elevated local strains continue to accumulate at the weakest spots, resulting in strut failure [38].

The deformable titanium layers in the implants were assumed to have deformed during insertion as the oversized implants were flush to the bone without gaps, indicating considerable deformation. This deformation was quantified by measuring the incurred volume reduction using post-insertion CT images. However, this (again) is an approximation as the exact value of the volume reduction is difficult to determine from a CT scan due to beam hardening and the partial volume effects [39].

All implants showed additional subsidence between the first and last compression up to 1800 N of the cyclic tests. In literature this is sometimes described as ‘bedding-in’ (or subsidence or migration) of the implant [40]. We postulate that the implants ‘settle’ during the first cycles in which some extra deformation takes place. The subsidence between the first and last compressions was comparable to the values found in literature [41]. Saffarini et al*.* have described subsidence values between 0.05 and 0.27 mm for cementless hemispherical cups [41]. Moreover, subsidence curves over time in the study of Saffarini et al*.* is consistent with the curves obtained during our experiments.

This study also has its limitations. First of all, using as-manufactured implants could have influenced the mechanical properties and strut failure during insertion of the implants. A recommended post-processing step is Hot Isostatic Pressing (HIP) [42]. For pure titanium (Grade 1), HIP treatment may further reduce the yield strength of the bulk material and increase its plastic deformation (ductility) [43]. This will translate to increased deformability and reduce the risk of strut failure in the deformed porous structures.

The second limitation is the use of Sawbones hemipelves. The inner solid foam that represents the trabecular bone is isotropic whereas the trabecular bone found in the human pelvis can be considered as largely transverse isotropic [44]. Furthermore, the yield strength of human pelvic trabecular bone is 3–10 times lower than the yield strength of the solid foam used in the Sawbones pelvises [28]. Also the acetabular defects created in the Sawbones are a simplified representation of the defects found in complex revision arthroplasty, which is a limitation of this study. That said, Sawbones are one of the best ways to evaluate implant designs at the conceptual design stage because their mechanical behaviour is highly reproducible (as opposed to cadaveric specimens), enabling different design concepts to be objectively compared with each other.

Another limitation is the stress–strain plot of the 3 × 3 × 3 mm unit cell size that did not show three, but two linear elastic sections (Fig. 7a). This was likely caused by lack of sufficient deformation in the least porous section (*i.e.*, 0.45 mm strut thickness), which did not deform under a 5 kN load and therefore an elastic modulus and compressive yield strength could not be estimated.

The most optimal unit cell size we considered was 4 × 4 × 4 mm with a 200 microns thick strut thickness, leading to a yield stress of 0.168 MPa that worked well within the simulated clinical setting. However, the implant design is significantly restricted once whole 4 mm unit cells are incorporated into the structure. In order to be able to effectively describe a volume with this ratio with a porous structure, one should ideally work with shorter struts with an even smaller thickness than 200 microns.. However, such thin structures are currently not possible with the most advanced additive manufacturing techniques [45].

Plastically deformable acetabular cups seems a technically viable solution for filling critical sized acetabular defects. The deformable layer will increase the load transfer through the acetabular cavity, as compared to flanges only. According to Wolff’s law, the increased load transfer stimulates bone to grow into the highly porous structure providing a strong secondary fixation and ultimately will preserve the cancellous bone in the deep acetabular zone [16, 17, 46]. In addition, the porous printed structure can be designed using architectures that lead to high expansion in the transverse directions, thereby filling gaps of the bone defects upon insertion. Whether this leads to better clinical outcomes cannot be supported from this proof of concept study and needs to be investigated further.

In conclusion, it is possible to realize primary implant fixation in simulated acetabular revision surgery with bone defects, using acetabular cups with an incorporated deformable titanium scaffold. However, additional research is needed to investigate the effects of the deformable titanium on actual real bone specimen.

### Supplementary Information

**Additional file 1.** 

**Additional file 2.**

**Additional file 3.**

## Figures and Tables

**Fig. 12:**
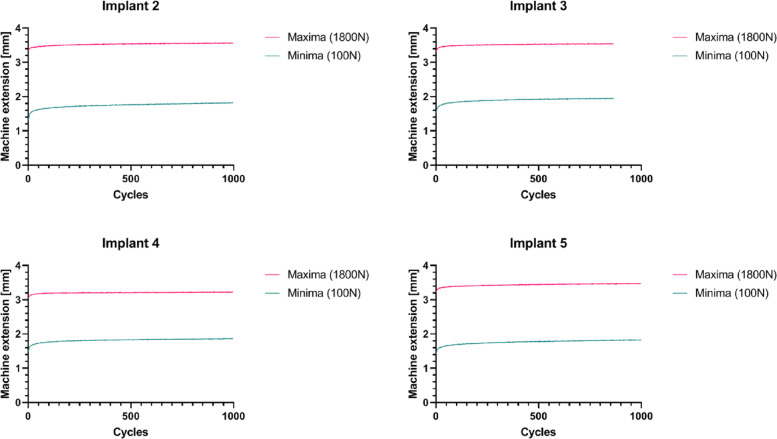
The cycles-extension curves for the cyclic test of implant 2, 3 and 4. The extension needed to reach 1800 N during the first compression is indicated by the red line. The relaxation to 100N is shown by the blue line1

## Data Availability

Not applicable.

## Abbreviations

SLM: Selective laser melting
BCC: Body centered cubic
CT: Computed tomography
SLS: Selective laser sintering
CAD: Computer-aided design
HIP: Hot isostatic pressing
